# 2825. Clinician Treatment Considerations and Decisions in Hypothetical Uncomplicated Urinary Tract Infection Patient Vignettes

**DOI:** 10.1093/ofid/ofad500.2436

**Published:** 2023-11-27

**Authors:** Jeffrey J Ellis, Kristin J Moore, Bonnie H K Bui, Jennifer Friderici, Maureen Carlyle, Carolyn K Martin, Noah S Webb, Megan E Luck, Darrian Proco, Kristin Kahle-Wrobleski

**Affiliations:** GSK, Collegeville, Pennsylvania; Optum, Eden Prairie, Minnesota; Optum, Eden Prairie, Minnesota; Optum, Eden Prairie, Minnesota; Optum, Eden Prairie, Minnesota; Optum, Eden Prairie, Minnesota; Optum, Eden Prairie, Minnesota; GSK, Collegeville, Pennsylvania; GSK, Collegeville, Pennsylvania; GSK, Collegeville, Pennsylvania

## Abstract

**Background:**

For uUTI, Infectious Diseases Society of America (IDSA) guidelines recommend empiric treatment (Tx) based on clinical presentation and patient (pt) factors (including age, allergies, drug resistance, recurrence history). This study aimed to understand the decision-

making process among clinicians treating pts with uUTI.

**Methods:**

US clinicians treating pts with uUTI completed an online cross-sectional survey collecting diagnosis and Tx decision-making information. Inclusion criteria were self-reported: currently practicing, having seen ≥1 uUTI pt(s) in the past month and ≥12 uUTI pts in the past year, with 2–25 years’ experience post-training. Survey items included clinician/practice characteristics and pt vignettes. Survey items probing diagnosis and Tx decision-making followed each vignette. Analyses were descriptive.

**Results:**

206 clinicians completed the survey (49% physicians, 33% nurse practitioners, 18% physician assistants); 71% practiced in primary care (Table 1). The proportion of clinicians who reported that their practice maintains guidelines for uUTI diagnosis and Tx was, respectively: for an initial uUTI, 32.5% and 45.6%; for recurrent/persistent uUTI, 38.8% and 39.8%. Pt experience with other therapies was selected by 52.9% of respondents as the most important characteristic in Tx selection; meanwhile, pregnancy (88.4%), allergies (84.0%) and prior compliance (78.6%) were frequently reported factors considered when making prescribing decisions. For vignette-specific Tx decisions, NTF and SXT were the predominant initial Tx selections; 86–97% of respondents reported they took antimicrobial resistance into consideration (Table 2). Though infrequent, Tx selection incongruent with IDSA guidelines was

observed, e.g., selection of same antibiotic that failed to resolve uUTI (vignettes 3 and 6). Clinical presentation and uUTI history were the highest ranked vignette-specific factors important to Tx decisions (Figure).

**Figure:**
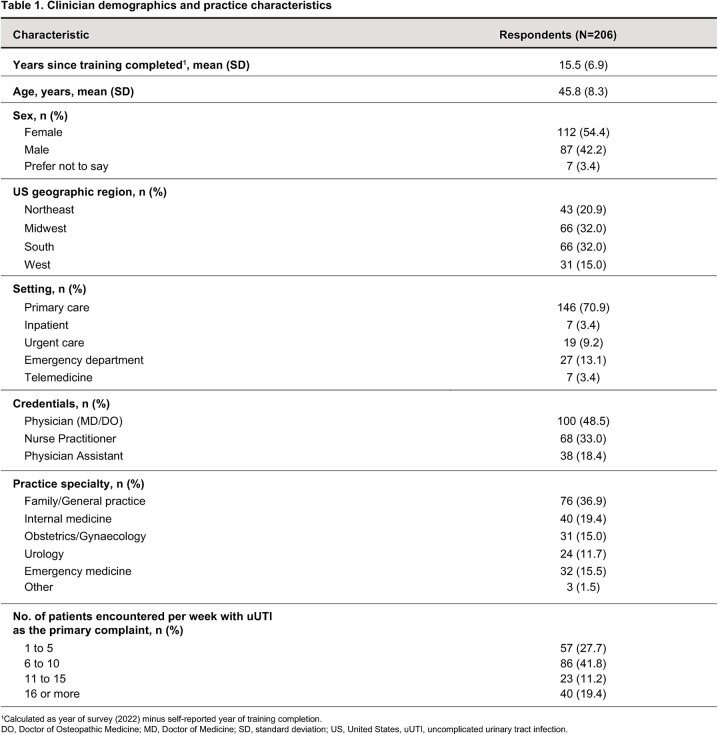

**Figure:**
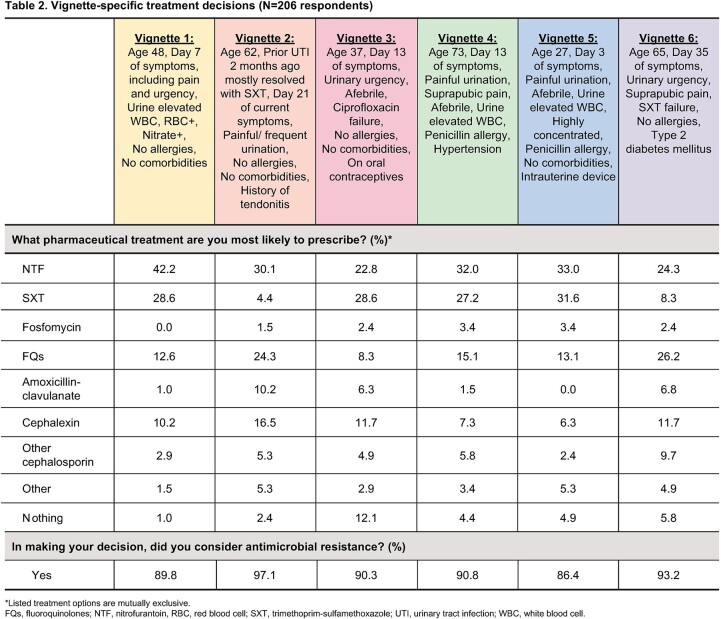

**Figure:**
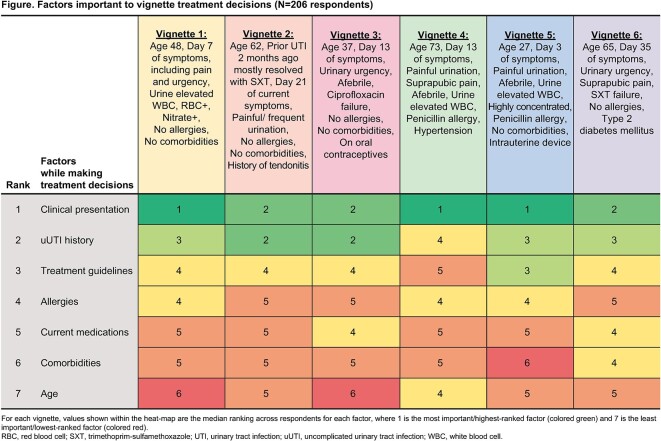

**Conclusion:**

Vignette Tx decisions did not always align with IDSA guidelines. Future work should compare decision-making processes of physicians and other clinicians and assess the impact of barriers to appropriate Tx selection, such as the absence of practice-maintained guidelines.

**Disclosures:**

**Jeffrey J. Ellis, PharmD, MS**, GSK: Jeffrey J. Ellis is an employee of, and shareholder in, GSK **Kristin J. Moore, PhD, MPH**, Optum: Kristin J. Moore is an employee of Optum, which received funding from GSK to conduct this study **Bonnie H.K. Bui, PhD**, Optum: Bonnie H.K. Bui is an employee of Optum, which received funding from GSK to conduct this study **Jennifer Friderici, MS**, Optum: Jennifer Friderici is an employee of Optum, which received funding from GSK to conduct this study **Maureen Carlyle, MPH**, Optum: Maureen Carlyle is an employee of Optum, which received funding from GSK to conduct this study **Carolyn K. Martin, MSW**, Optum: Carolyn K. Martin is an employee of Optum, which received funding from GSK to conduct this study **Noah S. Webb, PhD**, Optum: Noah S. Webb is an employee of Optum, which received funding from GSK to conduct this study **Megan E. Luck, PharmD**, GSK: Employee of, and shareholder in GSK **Darrian Proco, PharmD**, GSK: Darrian Proco is an employee of, and shareholder in, GSK **Kristin Kahle-Wrobleski, PhD**, GSK: Kristin Kahle-Wrobleski is an employee of, and shareholder in, GSK

